# Acceptability and efficacy of intra-rectal quinine alkaloids as a pre-transfer treatment of non-per os malaria in peripheral health care facilities in Mopti, Mali

**DOI:** 10.1186/1475-2875-6-68

**Published:** 2007-05-22

**Authors:** Mahamadou A Thera, Falaye Keita, Mahamadou S Sissoko, Oumar B Traoré, Drissa Coulibaly, Massambou Sacko, Valerie Lameyre, Jean Pascal Ducret, Ogobara Doumbo

**Affiliations:** 1 Malaria Research and Training Centre, Department of Epidemiology of Parasitic Diseases (DEAP)/Faculty of Medicine, Pharmacy and Odonto-Stomatologie (FMPOS), University of Bamako, Bamako, Mali; 2 Mali National Malaria Control Program, Ministry of Health, Bamako, Mali; 3 Impact Malaria, Sanofi-aventis, Gentilly, France; 4 Regional Office of World Health Organization WHO/AFRO, Local Office of Mali, Bamako, Mali

## Abstract

**Background:**

The acceptability and efficacy of a new kit with a new formulation of quinine alkaloids designed for the intra-rectal administration in the treatment of non-per os malaria was assessed in the peripheral health care system of Mopti, Mali.

**Methods:**

A single-arm trial was conducted from August 2003 to January 2004. An initial dose of diluted quinine alkaloids (20 mg/kg Quinimax^®^) was administered by the intra-rectal route to children with presumptive non per-os malaria at six peripheral heath care centres. The children were then referred to two referral hospitals where standard inpatient care including intravenous route were routinely provided. A malaria thick smear was done at inclusion and a second malaria thick smear after arrival at the referral facility, where a more complete clinical examination and laboratory testing was done to confirm diagnosis. Confirmed cases of severe malaria or others diseases were treated according to national treatment guidelines. Cases of non per-os malaria received a second dose of intra rectal quinine alkaloids. Primary outcome was acceptability of the intra rectal route by children and their parents as well as the ease to handle the kit by health care workers.

**Results:**

The study included 134 children with a median age of 33 months and 53.7% were male. Most of the children (67%) and 92% of parents or guardians readily accepted the intra-rectal route; 84% of health care workers found the kit easy to use. At the peripheral health care centres, 32% of children had a coma score ≤ 3 and this was reduced to 10% at the referral hospital, following one dose of intra-rectal quinine alkaloids (IRQA). The mean time to availability of oral route treatment was 1.8 ± 1.1 days. Overall, 73% of cases were confirmed severe malaria and for those the case fatality rate was 7.2%.

**Conclusion:**

IRQA was well accepted by children, their parents/guardians and by the health workers at peripheral health facilities in Mopti, Mali. There was also a quick recovery from deep coma and a reduced case fatality rate in severe malaria.

## Background

Clinical malaria can be severe right from its onset. This is strongly related to individual susceptibilities [1] and often caused by delayed access to appropriate treatment. Delayed access to appropriate treatment is known to lead to increased mortality related to severe malaria [2,3].

In developing countries such as Mali, West Africa, the health care system is organized to allow treatment of severe malaria at the hospital level. However, most cases of malaria occur in rural areas far away from any hospital. Although local care providers exist in most remote areas they are not trained to adequately manage severe malaria episodes. Combined with others factors [4,5], this contributes to a greater delay in accessing adequate treatment and to the increase of the incidence of severe disease. The treatment of severe malaria episodes solely at the level of hospital bears the risk of increasing malaria case fatality rates. The fact that most of deaths recorded at hospitals occurred during the first 24 hours after admission [6,7] highlights the need for earlier actions to reduce case fatality rates.

Quinine is a critical drug widely used for the treatment of severe malaria [8]. Despite few cases were *P. falciparum *strains with decreased sensitivity to quinine were reported [9], the drug is recommended by most malaria control programmes in sub-Saharan Africa.

The use of intra rectal route for administering quinine has a proven efficacy in the treatment of uncomplicated malaria in several studies when compared to intra-muscular or intravenous routes [10]. Intra rectal quinine (IRQA) was also effective in the treatment of severe malaria and is an alternative to the intra muscular route, in situation where intravenous injection cannot be performed [11]. Initial trials have used the bichlorhydrate salt, with pH 2, which was aggressive for the anal epithelium and had a reduced bio-availability [12]. Recent studies have used a new gluconate salt formulation at pH 4.5. The increased pH resulted into a greater bio-availability and better local tolerability [13].

The intra rectal route is commonly used in some Malians villages by mothers for the treatment of others pathologies and by health workers to treat seizures with diazepam.

The Malian National Malaria Control Program (MNMCP) decided since 2002 to promote malaria case management within communities with the aim to allow earlier treatment of malaria clinical episodes. For severe malaria, the MNMCP chose an emergency one or maximum two doses of intra rectal quinine at periphery before referral. The doses would be given outside health care services by members of the communities (so called "agent relais" (AR) *i.e*., community health workers), under the supervision of the health care personnel working at the primary contact point between population and health care services. In Mali, the first contact with health care services is organized to occur at the community-based health care centre. A step-wise approach was adopted to introduce quinine usage outside health care services. In a first step, this pilot study of the acceptability of the IRQA was designed, to assess how the peripheral health care personnel and the target population will accept the approach in the district of Mopti. In a second step would be evaluated the acceptability and efficacy of the intra-rectal route when used by ARs. The final step would be the gradual scale-up of the approach to reach national coverage.

The results of the pilot study that assessed the acceptability of the intra rectal gluconate salt quinine given at periphery by health care agents as an alternative early pre-transfer treatment strategy of severe malaria in Mopti are reported in this paper.

## Methods

### Study site

The study was conducted in the district of Mopti located in central Mali, an area of seasonal and intense malaria transmission during the rainy season from July to November. *Anopheles gambiae s.l*. is the main vector and at peak transmission the entomologic inoculation rate (EIR) can reach, 22 infective bites per person and per night [14]. *Plasmodium falciparum *carriage rate was 40–80% and malaria accounted for 30% of overall cause of mortality *i.e*. the first child killer in the area [14].

Health services are organized in three levels of care in the region of Mopti. At periphery, the first point of contact with population is the community-based health care centre, run by a nurse or a physician and equipped to provide clinical care with no laboratory support. At the second level, district hospitals served as referral structures for the community-based health care centres. Each district hospital covers a population close to 150,000–200,000 inhabitants and provide medical and surgical services including gynaecology and obstetric with clinical laboratory. At the third level, the regional hospital located in the city of Mopti, provides medical, pediatric, general and specialized surgical care (including obstetric, ophthalmology and traumatology) and has capacity for laboratory diagnosis and X-rays. Despite its regional vocation the regional hospital of Mopti (HRM) mainly served as referral centre for the city of Mopti and its surroundings. Our study focused on community-based health centres that refer usually to the HRM and to the district hospital of Sevare.

The study was conducted among children attending six peripheral community-based health care centres in four villages (Medina Coura, Fatoma, Soufroulaye, Socoura), located at a maximum distance of 12 km from the city of Mopti. In addition two urban community-based health care centres both located in the city of Mopti were included. Children were given an initial dose of IRQA if they fulfilled the criteria for entry in the study and were either referred to the HRM in the case of children from Medina Coura and the two urban community health care centres, or to the referral health care centre of Sevare for children from Fatoma, Socoura and Soufroulaye.

Before the study started, the personnel of the selected health care centres were trained by the MNMCP and the team of researchers to standardize their diagnosis of malaria and to use the IRQA. In addition, the study protocol and procedures were explained. The personnel were instructed to recruit cases from the population that routinely attended their centres for care and that fulfilled the entry criteria into the study.

### Study patients

Children presenting spontaneously at peripheral health care centres and looking for care were eligible. Children were included if they weighed between 5 and 25 kg, were diagnosed on a clinical basis as a non-per-os malaria episode, and if their parents/guardians gave consent after receiving information about the study. The following exclusion criteria were applied: refusal of parents, association with others evident and severe clinical conditions such as severe malnutrition or meningitis, diarrhoea, pre-existing anal pathology such as prolapsus, rectitis, anal fissure, chronic diarrhoea, contra-indication to quinine and uncomplicated malaria episode.

### Study design

After an initial clinical assessment, including weighing the children, blood was collected by finger prick for a malaria thick smear which was not read immediately. A dose of 20 mg/kg of diluted quinine was administered by rectal route. Children were observed for 30 minutes and in the case of early expulsion of the solution, they received a further half dose. Afterwards they were transferred to the referral health care structures, in Sevare or in Mopti. Study team offered transportation for parents who needed it. At this level a more complete clinical and laboratory assessment was done. Finger prick blood was collected for a second malaria thick smear, determination of blood sugar and haemoglobin level. Malaria smears were read then and children were classified into two groups: confirmed cases of malaria and other diagnosis of children severe febrile illness (*i.e*., meningitis or acute respiratory infection). Children with *P. falciparum *positive slides and the criteria for severe malaria defined by the World Health Organisation (WHO) [8] were considered confirmed cases of severe malaria and were treated with intravenous quinine. Confirmed non-per os cases of malaria were given a second dose of IRQA plus a half dose in case of early expulsion. Children diagnosed with others diseases were treated according to national guidelines. All children were closely followed until the oral route was feasible and the clinical symptoms resolved.

### Study drug

Quinine was used in the form of an emergency paediatric kit provided by Sanofi-aventis, France. The solution of Quinimax^® ^is composed of 96.1% of quinine, 2.5% of quinidine gluconate, 0.63% of cinchonine and 0.67% of cinchonidine hydrochloride with a pH at 4.5. The mixture of alkaloids was constituted with the aim to reproduce as closely as possible, the composition of the natural bark of quinquina.

The kit was constituted of a 4 ml vial of Quinimax^®^, a vial of 13.5 ml purified water and a round-ended syringe specially designed for rectal administration. The syringe was marked according to weight and was validated to ensure that the amount of diluted Quinimax^® ^corresponded to a given weight of the child and had a content of quinine corresponding to the dose of 20 mg/kg for that specific weight. The overall content of the 4 ml vial was 500 mg gluconate alkaloids of quinine. Just before use, the content of the vial of Quinimax^® ^was diluted with the 13.5 ml of purified water giving a final dilution of 28.6 mg/ml. Only the amount of diluted solution corresponding to the weight of the child was withdrawn from the vial into the syringe. The syringe was marked with kilogrammes instead of millilitres in order to avoid the need for a calculation of dose at peripheral level and to facilitate the use of the paediatric kit.

### Definitions used

Severe malaria was defined as episodes of fever associated with *P. falciparum *parasite in peripheral blood and one of the following criteria: haemoglobin ≤ 5 g/dl, parasite density ≥ 100,000/μl, blood sugar ≤ 40 mg/dl, respiratory distress, seizures in the last 24 hours, coma, or jaundice.

Severe febrile illness associated with persisting vomiting and inability to drink or eat, in the absence of evident clinical signs or symptoms for a specific diagnosis, was considered as a non per os malaria episode.

### Outcome measurements

Study primary outcome was the acceptability of the intra-rectal route by children, their parents or guardians and by health care centres personnel. Acceptability of children was assessed by the presence of particular reactions during drug administration, such as pain (manifested by screaming during administration or verbal expression for older children); presence of particular reactions shortly (30 minutes) after drug administration: local pruritis, pain, false sensation of stools for older children/important screaming for younger children/for all children presence of diarrhoea, blood in stools, inflammation of anal mucosis. These particular reactions were assessed by health care centres personnel at periphery after first administration of IRQA and at the referral level after the second administration. Anal margin and stools aspects were directly observed.

Parents or guardians acceptability was assessed by interview and asking them how they would feel if another administration of the intra rectal route was proposed to treat their children.

Health care centre personnel acceptability was assessed by asking them to fill in the case report form whether it was easy or uneasy to prepare the solution for intra rectal administration, to read the marks on the syringe for dosing the amount of solution to be given and to administer/re administer (in case of early expulsion) the solution.

Secondary outcomes were time to recovery of oral route and time to clearance of clinical signs/symptoms. These outcomes were measured twice a day for 7 days after inclusion into the study.

### Data collection and quality control

Before the study started in June 2003, all clinical personnel involved in collecting data and administering IRQA were trained to comply with standardized study procedures for obtaining informed consent, clinical examination, laboratory testing, filling in of case report forms, administration of IRQA using the emergency paediatric kit. Subsequently clinical, behavioural and laboratory data collected during the study were noted directly into standardized case report forms. Case report forms were verified on a daily basis by the study supervisor and three monitoring visits were conducted during the study to ensure quality of the data and compliance with protocol.

### Data analysis

Data from standardized case report forms were double-entered with Microsoft ACCESS and database was reconciled. Analysis was done using SPSS 11.0 (Chicago, Illinois). Descriptive analysis was done, computing percentages and means on main endpoints with 95 confidence intervals. Additional analyses included comparisons of proportions using Pearson chi-square and Yates corrections or Fischer exact tests where indicated. Mc Nemar chi-square was used for paired data. Means were compared using Student T test with adjustment to account for repeated measurements on the same individual. ANOVA was used to compare means. A difference was considered significant if *p-value *was less than 0.05.

### Ethical issues

The study protocol was submitted to and approved by the Faculty of Medicine and Pharmacy Institutional Review Board before the study commenced. Informed consent was obtained from parents/guardians of children prior to their enrolment. The same quality of care was offered to children in case the parents refused to be part of the study.

## Results

Overall 134 patients were enrolled from June 2003 to January 2004. One child died before arriving at the referral centre; two others died, one forty minutes and the second three hours and twenty two minutes after arriving at the referral centre. Out of 133 children that arrived at the referral centre, 97 (73%) had malaria smear positive for *P. falciparum*. Thirty children with negative malaria smears were diagnosed with others diseases. Sex ratio was 1.13 for male. The mean age of all children was 36.9 months. The age of the youngest child was one month and the age of the oldest was 144 months. Mean weight of children was 11.8 kg with a minimum of 5 kg and a maximum of 23 kg. All children included received a first dose of IRQA. Early expulsion of the product after first administration occurred in 23.1% of the children and required re-administration of a half dose. A second dose was administered to 35.1% of the children at the referral unit. For these, the proportion of early expulsion increased significantly to 40.4% (n = 47, *p = 0.023*).

### Acceptability of the intra rectal route

After the first administration, 32.8% of children expressed specific reactions (Table 1). Some of the children cried and were agitated just prior to administration. Few parents (8.2%) were surprised and one parent said that he has no choice face to medical prescription. Most of the parents would agree to have a second dose of IRQA given to their child if needed. After a second administration of intra rectal Quinimax^®^, 10.6% of children had a specific reaction consisting of crying and contraction of back muscles prior to administration (Table 1).

**Table 1:** Acceptability of children and their parents/guardians

	All children (N = 134)		Positive thick smear (N = 97)	
	
After 1^st ^Intra rectal administration	*%*	95% CI	*%*	95% CI
Child reaction	*32.84*	25.29–41.12	*34.02*	25.12–43.86
Parents reaction	*8.21*	4.39–13.82	*7.21*	3.21–13.76
Parents accepting a new shot	*96.27*	91,93–98.62	*94.84*	88.95–98.09
	
	All children (N = 47)		Positive thick smear (N = 35)	
	
After 2^nd ^Intra rectal administration	*%*	95% CI	*%*	95% CI

Child reaction	*10,63*	4,00–22,01	*8,57*	2.23–21.58
Parents reaction	*2,13*	0,11–10,02	*2,85*	0,14–13,29
Parents accepting a new shot	*97,87*	89,95–99,89	*97,14*	86,71–99,86

Overall acceptability of IRQA was high after a second administration.

The majority of health care personnel found the emergency kit easy to store to prepare and to use. The difficulties evoked by 14.9% of the personnel during administration of the intra rectal route were due to children's agitation during the first administration. At the second administration, only 2.1% of the personnel, 7 times less, reported such difficulties, due to children crying and contracting back muscles during administration (Table 2).

**Table 2:** Easiness of the handling of the paediatric kit by health care personnel

	Peripheral care unit (N = 134)		Referral care unit (N = 47)	
	*%*	95% CI	*%*	95% CI
	
% of personnel declaring administration is easy	*85.07*	78.27–90.37	*97.87*	89.95–99.89
% of personnel declaring storage is easy	*100*	-	*100*	-
% of personnel declaring preparation is easy	*100*	-	*100*	-
% of personnel declaring reading of weights on syringe is easy	*100*	-	*100*	-

According to health care personnel the paediatric kit was easy to use.

### Clinical tolerance of the IRQA

The most common clinical symptoms presented by children following IRQA was the emission of liquid/semi liquid stools (Table 3). Paired analysis showed a significant three-fold increase in the incidence of liquid/semi liquid stools after one administration of IRQA. A more important increase was observed after the second intra rectal shot: but this was not associated with more signs of dehydration or more abnormalities of the anal margin. Among children with skin marks of dehydration (n = 21) at the referral health care level, 71.4% had liquid stools that started after the intra rectal administration. Observed abnormalities of the anal margin consisted of local inflammation in three children and one case of incipient prolapsus, at the referral health care unit. All these children had a history of diarrhoea in the previous 48 hours. Parents of one child reported presence of blood in stools but the evidence did not support this when referral health care personnel inspected stools. Abnormalities of the anal margin and presence of blood in the stools reported at the peripheral health care units were not confirmed by more experienced paediatric physicians after referral.

**Table 3:** Clinical tolerance after one or two administrations of IRQA.

After 1^st ^Intra rectal administration	Peripheral care unit (N = 130)	Referral care unit (N = 130)	
	
	*%*	*%*	*p-value*
Skin with dehydration marks	*3.8*	*16.2*	*< 0.01*
Abnormal anal margin	*2.3*	*3.1*	*ns*
Blood in stools	*0.8*	*0.8*	*ns*
	
	N = 115	N = 115	
	
Liquid/semi liquid stools	*25.2*	*75.7*	*< 0.01*

			
	After 1^st ^dose	After 2^nd ^dose	
	N = 47	N = 47	
	
	*%*	*%*	*p-value*

Skin with dehydration marks	*4.3*	*6.4*	*ns*
Abnormal anal margin	*2.1*	*-*	*ns*
Blood in stools	*0.8*	*0.8*	*ns*
Liquid/semi liquid stools	*19.1*	*44.7*	*< 0.01*

More children had liquid/semi liquid stools after the first administration of IRQA. The second administration was better tolerated.

### Efficacy of IRQA

Overall (n = 124), oral drugs could be administered after 1.8 ± 1.3 days, the minimum being one day and the maximum seven days. Confirmed cases of malaria with positive thick smear that survived (n = 90) had oral route available after 1.6 ± 0.9 days, with a minimum of one day and a maximum of five days. The frequency of clinical symptoms such as coma with Blantyre score ≤ 3 significantly decreased at the referral health care unit after IRQA (Table 5). Overall a case fatality rate of 7.46% 95% CI [3.85–13.89] was observed. Out of the ten deaths, seven were associated with signs of respiratory distress. For six cases biologic parameters were determined and among those three had blood sugar < 80 mg/dl and hemoglobin ≤ 5 g/dl. At inclusion 20% of children had low blood sugar level and 90% had mild anaemia (haemoglobin < 11 g/dl).

## Discussion

From August 2003 to January 2004 we assessed acceptability of IRQA as treatment of non per os malaria in the Mopti region, Mali. The framework of routine care offered at periphery according to the paediatric disease management policy of the Malian Ministry of Health was used.

This implementation study showed that administration of IRQA at peripheral health care centres in Mali was accepted by 95% of parents and 67% of the children. In majority, the personnel (85%) found the kit easy to use. The most common adverse reaction after administration of IRQA was emission of liquid stools in 75.7% of children after the first shot. We observed a case fatality rate of 7.5%.

Children presenting spontaneously at peripheral health care units were included. In such rural areas, attending the health care facility happened when all more affordable means to treat have been used unsuccessfully [5]. This might have favoured inclusion of more severely ill children in our study.

The diagnosis of severe malaria at periphery was based on symptoms and clinical signs only according to national guidelines. After referral, almost 30% of cases had negative blood smear and were diagnosed with others conditions.

In few cases, inclusion criteria were not strictly observed and some children that had a history of diarrhoea were included. Abnormalities of anal margins were reported in these children.

The study might have been biased to include children with less survival chances than cases of classic non per-os malaria, in which IRQA could be more efficacious and better tolerated. But the study population also reflects the reality of patients to whom IRQA will be administered in routine circumstances. In routine condition, the likelihood of strict adherence to definitions of non per-os malaria cases is rather low.

IRQA was well tolerated. Liquid and semi-liquid stools were the most common adverse reactions observed. A quarter of patients had liquid stools after first administration and when they reached the referral care centre the proportion increased to three quarters. In previous trials much lower proportion of liquid stools were reported [15,16]. Such difference can be explained by the differences in disease severity and clinical presentation at inclusion. Emission of liquid stools was associated with diminution of body fluids observed by signs of dehydration on patients' skin. No further impact on child survival was observed and stools became normal after 25–48 hours. In Gabonese children, severe malaria was associated with depletion of total body water although, as observed in Mopti, the diminution of body fluids did not correlate with severity of disease [17]. In addition a second administration of IRQA given to 47 children caused less frequent liquid stools and no impact on body fluids. This second dose was better tolerated than the first one.

In cases where abnormalities of anal margins were reported, the children had anal mucosis injured by previous episodes of diarrhoea. It emphasizes the need to respect contra indications of intra rectal route, *i.e*, to avoid cases of diarrhoea and lesion of the anal region. In summary the tolerance profile shown from this study favour the recommendation to further use IRQA in the treatment of severe malaria when the intravenous route is not available.

Health care personnel at peripheral and regional referral health care unit levels unanimously considered the use of the kit easy. This will facilitate the subsequent large-scale implementation of the pre-transfer one or two doses of IRQA in remote and resource poor areas of Mali under the auspices of the National Malaria Control Programme.

The sharp reduction in the severity of coma following one dose and the low case fatality rate observed in such biased study population confirmed the known efficacy of the IRQA. The role of confounding factors related to the single-arm design is balanced to some extent by the very high case fatality rate observed in untreated cases of severe malaria. Our observed case fatality rate,(7.5%) is close to data reported from Niger [18]. Higher case fatality rates (16–30%) were observed in similar studies in African hospitals [18,19], while others have reported case fatality rates as low as 3.5% [6] or 7–13% [7,20]. The above underlines the difficulties linked to the clinical management of severe malaria. Studies have shown that most deaths from severe malaria occurred in the first 24 hours after the onset of the disease [6]. In this study too, 7 out of 10 deaths occurred before 24 hours of the child's inclusion. The classical treatment is based on intravenous quinine administered with saline of glucose solution. The Gabon study [17] showed that administration of fluids may be harmful and finally the optimal treatment for severe malaria remains to be codified.

In rural areas in Africa, the rectal route is becoming a promising alternative. It effectiveness doesn't require co -administration with fluids. When implemented at the periphery of a weak health care system, as in Mopti, it reduces the delay to an effective antimalarial treatment. Rectal arthemeter has also proven to be well tolerated and effective in the treatment of severe malaria [21]. However, quinine has the advantage of being actually available and known by health care workers and mothers in most Malian rural areas. Such availability associated to its efficacy and local tolerance make the intra rectal route for treatment of severe malaria by quinine a precious public health tools with potential to rapidly decrease the burden of malaria.

## Conclusion

In summary this study showed that IRQA is safe as long as contra-indications to its use are respected and efficacious for the initial treatment of severe malaria at peripheral health care level in Mali. The study supports the further implementation of the step-wise approach to introduce quinine usage outside health care services in Mali.

## Authors' contributions

OK Doumbo, MA Thera, V Lameyre and JP Ducret designed the study. OK Doumbo, MA Thera, V Lameyre and M Sacko developed the protocol. MA Thera directed the overall conduct of the study and the data analysis. F Keita and OB Traore conducted the study in the field and contributed to data analysis. MS Sissoko analysed the data and monitored the study. D Coulibaly monitored the study. MA Thera, OK Doumbo, V Lameyre, M Sacko, F Keita, MS Sissoko, OB Traore D Coulibaly and JP Ducret wrote the paper. All authors read and approved the final manuscript.

**Table 4:** Repartition of the ten deceased children by score of coma at inclusion and at the referral health care unit, parasite density, haemoglobin level blood sugar at inclusion and time elapsed from inclusion to death

Coma score at peripheral unit	Coma score at referral unit	Parasite density	Blood sugar (mg/dl)	Haemoglobin (g/l)	Time to death (in hours & mn)
1	3	93330	102	8,1	50 h30 mn
2	3	66600	36	5,3	36 h45 mn
1	2	211300	45	8,1	10 h30 mn
2	0	52350	89	10,5	14 h50 mn
5	1	9700	131	5,0	49 h55 mn
1	2	0	48	5,3	40 mn
0	-	97775	-	-	21 h25 mn
0	-	0	-	-	3 h22 mn
0	-	12375	-	-	1 h30 mn
2	-	-	-	-	1 h

Only three deaths occurred after 24 hours from the child's inclusion into the study.

**Table 5:** Proportion of children with signs and symptoms at peripheral and referral health care units after IRQA (paired data)

All children	Peripheral care unit (N = 130)	Referral care unit (N = 130)	
	
	*%*	*%*	*p-value*
Fever	*79.2*	*70.0*	*ns*
Convulsions	*77.7*	*74.6*	*ns*
Vomiting	*71.5*	*56.9*	*0.05*
Coma Blantyre score ≤ 3	*54.6*	*20.8*	*< 0.01*

Confirmed cases of malaria	(N = 95)	(N = 95)	
	
	*%*	*%*	*p-value*

Fever	*87.4*	*73.7*	*< 0.01*
Convulsions	*76.8*	*74.7*	*ns*
Vomiting	*70.5*	*58.9*	*ns*
Coma Blantyre score ≤ 3	*58.9*	*23.2*	*< 0.01*

All signs and symptoms improved from peripheral health care unit to referral hospital, even if statistical significance was not achieved for convulsions.
